# Role of Catheter Ablation in Arrhythmogenic Right Ventricular Dysplasia^1^

**Published:** 2005-04-01

**Authors:** Johnson Francis, Guy Fontaine

**Affiliations:** * Department of Cardiology, Medical College, Calicut, Kerala, India; † Service de rythmologie, Institut de cardiologie, hopital La Pitie-Salpetriere, Paris, France

**Keywords:** Arrhythmogenic Right Ventricular Dysplasia, Ventricular Tachycardia, Catheter Ablation

## Abstract

Arrhythmogenic right ventricular dysplasia/cardiomyopathy is a disorder characterized by frequent ventricular tachycardia originating from the right ventricle and fibro-fatty replacement of right ventricular myocardium. Though the disorder was originally described during surgical ablation of refractory ventricular tachycardia, catheter ablation of tachycardia is one of the options for patients not responding to anti arrhythmic agents. Direct current fulguration was used in the initial phase followed by radiofrequency catheter ablation. In the present day scenario, all patients with risk for sudden cardiac death should receive an implantable cardioverter defibrillator. Radiofrequency catheter ablation remarkably reduces the frequency of defibrillator therapies. Direct current fulguration can still be considered in cases when radiofrequency ablation fails, though it requires higher expertise, general anesthesia and carries a higher morbidity. Newer mapping techniques have helped in identification of the site of ablation. In general, the success rate of ablation in arrhythmogenic right ventricular dysplasia is less than in other forms of right ventricular tachycardias like right ventricular outflow tract tachycardia.

## Introduction

Arrhythmogenic right ventricular dysplasia/cardiomyopathy (ARVD) is a disorder characterized by fibro-fatty replacement of the right ventricular myocardium, frequent ventricular tachycardia originating from the right ventricle and right heart failure. It was originally described by Fontaine et al during surgical ablation of refractory ventricular tachycardia [1]. The first case of ARVD underwent surgical ablation in October 1973 with a simple incision made at the site of origin of ventricular tachycardia (VT). This was successful to prevent recurrence of the arrhythmia and contributed to the identification of this disease [2]. The first clinical series of 24 adult cases was published by Marcus et al in1982 [3]. The refractory ventricular arrhythmias of ARVD has always been a challenge for the clinician. Catheter ablation has been tried in spite of the justifiable fear of perforation of the dysplastic ventricle. This short review aims at bringing together the available literature on catheter ablation in ARVD.

## Therapeutic Options in ARVD

Though historically the original description of ARVD was during surgical ablation, pharmacological therapy was the initial mode of treatment in most cases. Surgical ablation by right ventricular disconnection was resorted to in resistant cases [4]. Peroperative cryoablation of 8 cases were reported by Isobe from Japan [5]. None of the eight patients died during a mean follow up of 3.25 year. VT recurred in two patients and a new VT was seen in another patient. Endocavitary electrode catheter ablation using direct current shocks of 100 to 320 J was another mode of therapy which was tried in that period [6]. Nowadays, implantable cardioverter defibrillators (ICD) are being recommended more often to cover the risk of sudden cardiac death (SCD) in ARVD. Even then, patients may need pharmacological therapy to reduce the number of shocks. Patients having recurrent sustained VT while on optimal medical therapy are candidates for catheter ablation.

## Catheter Ablation in ARVD

The initial reports on catheter ablation in ARVD were using direct current fulguration [6-8]. One of the earliest series of fulguration in ARVD was that of 13 patients who were treated with shocks ranging from 160 to 280 J [9]. Single or multiple shocks were required in up to three sessions. There were two deaths and four of the 11 survivors required antiarrhythmic treatment following the fulguration therapy. The mean follow up was 45 months. With the advent of radiofrequency catheter ablation, direct current fulguration went out of vogue. Fontaine et al has suggested that fulguration should still be tried if radiofrequency ablation is not successful [10]. In their 16 year experience of ablation in ARVD, the effectiveness of  radiofrequency was less than 40% in the first session. At same time, fulguration is effective in the same session after ineffective radiofrequency ablation. Complications have disappeared since the use of soft and steerable ablation catheters. This work also classifies thoroughly the results of VT ablation alone and in combination with antiarrhythmic drugs, definition of relapses, etc. But the disadvantage of fulguration is that it requires expertise, general anesthesia and more than one session in half the patients.

## Radiofrequency Catheter Ablation

Radiofrequency catheter ablation for ARVD has been in use since early nineties [11]. It has been suggested that only patients with focal dysplasia are potential candidates for ablation [12].

Entrainment mapping can be used to characterize reentry circuits in ARVD to guide ablation [13-15]. The concept of concealed entrainment was first reported by Fontaine et al in 1989 [13]. This paper stresses the identification of the zone of slow conduction. The concept was originally found in a patient with ARVD. It was later extended to other forms of chronic VT (post-myocardial infarction), and more recently is used as a marker of the reentry pathway in re-entrant supraventricular tachycardias. Ellison et al mapped 19 VTs in 5 patients with ARVD. Radiofrequency current was applied to the 58 sites where pacing entrained the VT to assess acute termination, with only 22% success. Eight of the 19 VTs were rendered noninducible and three were modified to a longer cycle length. In two patients ablation at a single site abolished two VTs [14]. Harada et al did entrainment mapping in 8 VTs in 7 patients with ARVD. Radiofrequency applications were done at 31 sites identified by mapping and terminated 7 of them [15].

Endocardial mapping can detect abnormal fragmented electrograms with delayed potentials. Pacemapping confirms the ablation site by producing a QRS morphology identical to the clinical VT [16]. Recently non-contact mapping has been used to guide catheter ablation in ARVD [17]. The endocardial exit point was defined in all three ARVD patients and the diastolic pathway (earliest endocardial diastolic activity) was identified in one of them. Catheter ablation was completely effective in only one of the three. Reithmann et al used electroanatomic mapping of right ventricular endocardial activation as a guide for catheter ablation in patients with ARVD [18]. Both electroanatomic mapping and entrainment procedures were performed in 5 patients. Endocardial mapping during tachycardia demonstrated a focal activation pattern with radial spreading of activation from the site of earliest activation. The sites of earliest activation were in an aneurysmal outflow tract in two patients, at the border of aneurysms near the tricuspid annulus in two patients and at the apex of the right ventricle in one. Entrainment mapping showed that these were the exit sites of the reentrant circuits. The clinical VTs were noninducible in 4 of the 5 patients after catheter ablation. During a mean follow up of 7 months, the frequency of ICD therapies came down from 49 ± 61 episodes per month to 0.3 ± 0.5 episodes per month.

Three dimensional Real-time Positioning Management System (RPM) has also been used for guiding ablation in ARVD [19]. RPM uses sonomicrometry to determine the spatial location of the ablation catheter relative to two reference catheters positioned in the right atrium and right ventricle.

O'Donnell et al have highlighted the electrophysiological differences between patients with ARVD and right ventricular outflow tract tachycardia (RVOT VT) [20]. Though radiofrequency ablation is the first line treatment for symptomatic RVOT VT, the role is limited in ARVD. In their study they compared 33 patients with RVOT VT and 17 patients with ARVD. Re-entry was the mechanism of tachycardia in 80% of the ARVD group while 97% of RVOT VT had features of triggered automaticity. Partial or complete success was obtained only in 71% of patients with ARVD while complete success was obtained in 97% of RVOT VT. The recurrence rate was 48% in ARVD and 6% in RVOT VT.

Ablation of ventricular tachycardias in ARVD still remains a clinical challenge, though more and more cases are being reported in the literature [21-23].
